# Methylation markers *FAM19A4* and *miR124‐2* as triage strategy for primary human papillomavirus screen positive women: A large European multicenter study

**DOI:** 10.1002/ijc.33320

**Published:** 2020-10-21

**Authors:** Jesper Bonde, Arno Floore, Ditte Ejegod, Frederique J. Vink, Albertus Hesselink, Peter M. van de Ven, Anja Oštrbenk Valenčak, Helle Pedersen, Saskia Doorn, Wim G. Quint, Karl Ulrich Petry, Mario Poljak, Grazyna Stanczuk, Kate Cuschieri, Silvia de Sanjosé, Maaike Bleeker, Johannes Berkhof, Chris J. L. M. Meijer, Daniëlle A. M. Heideman

**Affiliations:** ^1^ Molecular Pathology Laboratory, Department of Pathology Hvidovre Hospital Hvidovre Denmark; ^2^ Self‐screen B.V Amsterdam The Netherlands; ^3^ HPV Research Group, Division of Pathology University of Edinburgh Scotland UK; ^4^ Department of Gynaecology and Obstetrics Klinikum Wolfsburg Germany; ^5^ Institute of Microbiology and Immunology University of Ljubljana Ljubljana Slovenia; ^6^ Infections and Cancer Laboratory Catalan Institute of Oncology (ICO) Barcelona Spain; ^7^ DDL Diagnostic Laboratory Rijswijk The Netherlands; ^8^ Amsterdam UMC, Vrije Universiteit Amsterdam, Pathology, Cancer Center Amsterdam Amsterdam The Netherlands; ^9^ Department of Obstetrics and Gynaecology Western Isles Hospital Scotland UK; ^10^ Department of Epidemiology and Biostatistics Amsterdam UMC, Vrije Universiteit Amsterdam Amsterdam The Netherlands

**Keywords:** biomarker, cervical carcinoma, cervical screening, DNA hypermethylation, human genome methylation, human papillomavirus

## Abstract

In human papillomavirus (HPV) cervical cancer screening, cytology is used as triage to counter the low specificity of HPV testing. VALID‐SCREEN is a EU‐multicenter, retrospective study conducted to evaluate the clinical performance of the FAM19A4/miR124‐2 methylation‐based molecular triage test as a substitute or addition to cytology as reflex testing of HPV screen positive women. FAM19A4/miR124‐2 methylation test (QIAsure Methylation Test) was evaluated in 2384 HPV‐positive cervical screening samples, from women 29‐76 years of age, derived from four EU countries. Specimens were collected in ThinPrep or SurePath media, HPV‐status, concurrent cytology, and histology diagnosis were provided by the parent institutes. The control population consisted of women with no evidence of disease within 2 years of follow‐up. A total of 899 histologies were retrieved; 527 showed no disease, 124 CIN2 (5.2%), 228 CIN3 (9.6%) and 20 cervical cancers (0.8%); 19 of 20 screen‐detected cervical cancers were found methylation‐positive (sensitivity 95%). Overall specificity of FAM19A4/miR124‐2 methylation test was 78.3% (n = 2013; 95%CI: 76‐80). The negative predictive value of hrHPV positive, methylation‐negative outcomes were 99.9% for cervical cancer (N = 1694; 95%CI: 99.6‐99.99), 96.9% for ≥CIN3 (95%CI: 96‐98), and 93.0% for ≥CIN2 (95%CI: 92‐94). Overall sensitivity for CIN3 using FAM19A4/miR124‐2 methylation test was 77% (n = 228; 95%CI: 71‐82). CIN3 sensitivity was uniform between centers independent of sample collection medias, DNA extraction methods and HPV screening tests. Being objectively reported compared to the subjectivity of cytology, equally performing across settings and screening methods, the *FAM19A4/miR124‐2* methylation constitute an alternative/supplement to cytology as triage method to be investigated in real‐life pilot implementation.

## Abstract 2

**What's new?**

In HPV screening for cervical cancer, cytology has been used to triage those women who are hrHPV‐positive. However, cytology is subjective, and skilled cytologists are often in short supply. A molecular test using specific biomarkers might offer better results. In this large European study, the authors found that a new standardized test for the methylation biomarker combination FAM19A4/miR124‐2 did indeed yield results that were equal to or better than triage by cytology, in determining which hrHPV‐positive women should be referred for colposcopy.

**Abbreviations**
ASCUS: atypical squamous cells of undetermined significance
CC: cervical cancer
CIN: cervical intraepithelial neoplasia
EU: European Union
*FAM19A4*: family with sequence similarity 19 (chemokine [C‐C]‐motif)‐like), member A4 (also known as *TAFA4*: TAFA chemokine like family member 4)
HC2: hybrid capture II
hrHPV: high‐risk human papillomavirus
HSIL: high grade squamous intraepithelial lesions
LBC: liquid‐based cytology
LSIL: low grade squamous intraepithelial lesions
*miR124‐2*: microRNA 124‐2
NHS: National Health Services (UK)
PPV: positive predictive value
qMSP: quantitative methylation‐specific PCR
TS: tumor suppressor

## 1 INTRODUCTION

With superior sensitivity for ≥CIN2 detection and an improved protection against cervical cancer, high‐risk human papillomavirus (hrHPV) based cervical screening has replaced or is scheduled to replace cytology as primary screening method in several countries with several other expected to follow shortly.^1^, ^2^, ^3^, ^4^, ^5^, ^6^, ^7^ To stratify hrHPV screen positive women with clinical disease from women with transient HPV infections, cytology‐based triage with or without HPV16 and HPV18 genotyping, is most commonly used. Yet, cytology remains a subjective method and, in many settings, trained cytologists are in short and ever dwindling supply. Implementation of HPV screening represents the largest transformation of secondary cervical cancer prevention since the introduction of cytology. However, to achieve the recently defined United Nations Sustainable Goal of reducing cervical cancer to a rare disease of 4 per 100.000 women, improved HPV screening strategies are required.^8^, ^9^, ^10^

Current HPV screening strategies are challenged by overdetection of clinically irrelevant, transient HPV infections which can result in unnecessary follow‐up visits, colposcopy overreferral and overtreatment. The lower specificity of HPV testing is today countered by reflex triage testing with (repeat) cytology, either with or without HPV 16/18 genotyping. Yet, cytology triage holds challenges too; in the Netherlands triage by abnormal cytology (threshold ≥ASCUS) in screening led to a ~3‐fold colposcopy referral increase with a ~6‐fold increase in detection of benign or CIN1 lesions compared to cytology as screening method.^11^ In addition, the quality of cytology is greatly reliant upon sample quality and remains a largely subjective analysis dependent upon skilled cytologists training where a heavy investment in quality assurance is essential. Also, the specificity of cytology will further decline because of the a priori knowledge of the HPV positive status,^12^ and as HPV vaccinated birth cohorts enter screening programs, the anticipation is a markedly reduced incidence of cytological abnormalities with a relative increase in low‐grade cytological abnormalities caused by nonvaccine HPV types. Worst case scenario, this will lead to diminished clinical performance of cytological abnormalities with resulting overreferral, overtreatment and lower efficacy of the screening programs.^13^ For HPV based screening to reach its full potential, new robust, objective molecular triage methods are desirable, preferably biomarker combinations which can precisely predict risk of progression to cancer and which are amenable to diverse biospecimens. Triage by molecular biomarkers can overcome some of these challenges as test outcomes are machine read and cut off values are established through clinical studies.

HPV infection alone is insufficient for progression to cervical cancer and additional genetic and epigenetic alterations in the host genome are pivotal parts in the oncogenic process.^14^, ^15^, ^16^ Here, silencing of tumor suppressor (TS) genes by DNA methylation of promotor regions is a hallmark of progressive oncogenesis. For the cervix, methylation levels of specific TS genes increase with severity of CIN grade peaking in cervical cancer.^17^, ^18^, ^19^ Such cancer‐like high methylation levels of TS genes are typically found in CIN3 and those CIN2 lesions associated with long‐term persistent HPV infections (≥5 years) with several genetic aberrations consistent with those found in cervical cancer. These so‐called advanced CIN lesions are presumed to have a high short‐term progression risk to cervical cancer in contrast to lesions with low methylation levels and no or only few genetic aberrations. Among biomarkers, silencing of tumor suppressor (TS) genes by DNA methylation of promotor regions is well established in cervical carcinogenesis and therefore a credible target for analysis.

Recently, the *FAM19A4/miR124‐2* ^19^, ^20^, ^21^, ^22^, ^23^ methylation test has been introduced as a commercial CE‐IVD triage test for screening and diagnostic purposes. As a triage analysis among HPV‐positive women, *FAM19A4/miR124‐2* methylation analysis displays a clinical sensitivity and specificity similar to cytology.^23^ Furthermore, a high intralaboratory and interlaboratory agreement across a range of cervical screening sample collection methods has been demonstrated.^24^ A large international study evaluating 519 cervical cancers from five continents showed uniformly that the *FAM19A4*/*miR124‐2* methylation assay detects 98.3% of all cervical cancers, independent of histotype, HPV genotype, geographical area and sample type.^25^ Two retrospective screening studies furthermore showed that HPV‐positive but *FAM19A4/miR124‐2* methylation‐negative women had a 14 year CIN3+ risk equal to that of concurrent negative cytology outcome yet an even lower risk for cervical cancer.^19^, ^20^ Thus, the *FAM19A4/miR124‐2* negative result provides safety that cervical cancer is not present by its high sensitivity for cervical cancer and its low long‐term risk for cervical cancer and advanced CIN lesions.

To investigate the clinical performance of a *FAM19A4*/*miR124‐2* methylation‐based triage in an HPV based screening setting, we conducted a large multicenter post hoc, retrospective, cross‐sectional study evaluating HPV‐positive women aged ≥29 years from different European screening settings covering the most common liquid‐based cytology (LBC) sample collection medias, DNA extraction methodologies and clinical validated HPV tests.

## 2 MATERIALS AND METHODS

### 2.1 Study design

A multicenter retrospective study was designed within the VALID‐SCREEN (EU‐HORIZON2020) framework to determine the clinical performance of the *FAM19A4/miR124‐2* methylation test for detection of histologically confirmed cervical cancer, CIN3, CIN2, and ≤CIN1 in hrHPV positive cervical specimens from women undergoing screening in Scotland, Denmark, Slovenia and the Netherlands. Data on hrHPV status and pathology diagnosis of the cervical cancer specimens were provided from the parent institutes. All participating institutes used clinically validated hrHPV DNA assays^26^, ^27^ to determine hrHPV status (Table 1). Cases were defined by histologically confirmed CIN2 or worse (≥CIN2) within a 2‐years follow‐up period. The control population consisted of women with no evidence of disease within 2 years of follow‐up. Women with ≥CIN2 detected after 2 years were excluded from the study.

**TABLE 1:** Sample collection media, DNA extraction method, cohort information by participating center

	Scotland	Slovenia	Denmark	The Netherlands	Overall
Sample collection media	ThinPrep	ThinPrep	SurePath	UCM	
DNA extraction platform	Qiacube	Biorobot EZ1^a^	MagNAPure 96	Hamilton Star Robot	
DNA extraction kit	QIAamp DNA mini	QIAamp DNA mini^a^	Roche DNA & VIRAL NA	NucleoMag tissue	
HPV assay	Cobas 4800 (Roche)	HC2 (QIAGEN) RealTi*m*e High Risk HPV Assay (Abbott)	Onclarity (BD) and CLART HPV2 (Genomica)	HC2 (QIAGEN)	
Cohort origin	PAVDAG^28^	Routine screening^29^	VALGENT4 cohort^30^ and routine screening samples	VUSA‐screen^31^	
HPV‐positive	281	1329	657	1303	3570
Excluded (N)^b^	107	326	76	405	914
Methylation tested (N)	174	1003	581	898	2656
Invalid methylation test (N)	13	75	157	27	272
Included in study (N)	161	928	424	871	2384
Mean age of included (y; range)	40.7	38.0	51.6	38.3	40.7
	(30.0‐61.0)	(30.0‐76.3)	(30.0‐65.0)	(29.0‐61.0)	(29.0‐76.3)
Methylation (N)	161	928	424	871	2384
QIAsure Methylation Test positive	60 (37.3%)	258 (27.8%)	127 (30.0%)	245 (28.1%)	690 (28.9%)
QIAsure Methylation Test negative	101 (62.7%)	670 (72.2%)	297 (70.0%)	626 (71.9%)	1694 (71.1%)
Cytology (N)	160	928	200	864	2152
No cytology	1	–	224^c^	7	232
NILM	108	716	45	670	1539
ASCUS	20	69	59	68	216
LSIL	12	71	41	42	166
HSIL	20	72	55	84	231
Histology (N)	138	236	302	223	899
No histology	24 (14.8%)	692 (74.6%)	122 (28.8%)	648 (74.4%)	1485 (62.3%)
No CIN	80 (49.4%)	59 (6.4%)	193 (45.5%)	32 (3.7%)	364 (15.2%)
CIN 1	18 (11.1%)	75 (8.1%)	34 (8.0%)	36 (4.4%)	163 (6.8%)
≤CIN1^d^	122 (75.3%)	826 (89.0%)	349 (82.3%)	716 (82.2%)	2012 (84.4%)
CIN 2	15 (9.3%)	42 (4.5%)	18 (4.2%)	49 (5.6%)	124 (5.2%)
CIN 3	20 (12.3%)	58 (6.2%)	49 (11.6%)	101 (11.6%)	228 (9.6%)
Cervical cancer	5 (3.1%)	2 (0.2%)	8 (1.9%)	5 (0.6%)	20 (0.8%)

^a^ Extraction was either done automatically with the Biorobot EZ1 (QIAGEN) or manually using the QIAamp DNA mini kit.

b Exclusion criteria: All centers; Women <29 y, Inadequate cytology at baseline, insufficient material for methylation testing. ≥CIN2 detected after 2 y of baseline. Center specific exclusion criteria: Scotland; ≥CIN2 detected after 2 y of baseline. Slovenia; Inadequate cytology, HPV vaccination recorded screening rounds, therapeutic intervention, No colposcopy data available, ≥CIN2 detected within 2 y. The Netherlands; No follow‐up data available until 2017, ≥CIN2 detected within 2 yof baseline.

c For Denmark, 224 samples were collected from routine HPV screening of women≥60 y. According to standard of care practice, HPV‐positive samples were not triaged by cytology, but directly referred to colposcopy.

d Calculated as total number of women in group with no histology, normal histology or CIN1 histology.

### 2.2 Study cohorts and sample processing

For each participating center, the cohort description, sample collection media, HPV screening assay and DNA extraction for methylation testing are summarized in Table 1. The inclusion criteria for samples from the different screening cohorts were (a) cervical scrapes derived from a screening cohort being hrHPV positive by a validated HPV screening assay; (b) containing sufficient material for cytology (where cytology was applicable) and valid methylation testing in concordance with the manufacturer's specification. Exclusion criterions were (a) women <29 years of age; (b) inadequate cytology at baseline (where cytology was applicable); (c) HPV negative or invalid test results at baseline, or insufficient material for methylation testing.

Sample selection was performed locally by the parent hospital or institution. Additional, center‐specific exclusion criteria for the Slovenian cohort were (a) HPV vaccination recorded at screening visit, (b) early therapeutic intervention, or (c) no colposcopy data available. For the Netherlands, no follow‐up data was available until 2017. A group of 224 samples from the Danish cohort did not have concurrent cytology as per Regional guidelines at that time. These Danish women, age 60 to 64 years, were under the screening exit program and were referred directly to colposcopy upon the hrHPV positive screening sample.

**Scotland**: Cervical samples were taken using the Cervex‐Brush (Rovers, Oss, the Netherlands) and collected in PreservCyt LBC (Hologic, Madison, Wisconsin). Cytology grading was according to British Society for Cytology and NHS Cervical screening program guidelines but translated into Bethesda nomenclature. Of 281 samples, a total of 161 samples were included in the final analysis. HPV testing was performed by Cobas4800 HPV test (Roche Molecular Systems, Pleasanton, California) as a consequence of the PaVDag evaluation).^28^ From the PreservCyt samples, 5% volume equalling 1 mL of the total volume was used for DNA extraction.

**Denmark**: All samples were collected using the Combi‐brush (Rovers, Oss, the Netherlands) in SurePath LBC media (BD Diagnostics, Durham, North Carolina). A total of 424 samples were included in the final analysis. HPV testing was performed by BD Onclarity HPV test (BD Diagnostics, Sparks, Maryland) or Genomica CLART HPV2 (Genomica SAU, Madrid, Spain). From the SurePath vial, 10% volume equalling 1 mL of the total cervical screening sample volume was used for DNA extraction.

**Slovenia**: Cervical screening samples were taken with the Cervex brush or Cytobush plus and collected in PreservCyt (Hologic, Madison, Wisconsin). A total of 928 samples were included in the final analysis. HPV testing was performed using the Hybrid Capture 2 assay (HC2; QIAGEN, Hilden, Germany) and the Abbott RealTi*m*e High Risk HPV Assay (Abbott Molecular, Chicago, Illinois). From the PreservCyt samples, 5% volume equalling 1 mL of the total volume was used for DNA extraction.

**The Netherlands**: Cervical samples were taken with a cytobrush and collected in 1 mL UCM (QIAGEN). Cytology grading was translated into Bethesda nomenclature. A total of 871 HPV‐positive samples were included in the final analysis. HPV testing was performed using HC2 (QIAGEN). From the UCM sample, 10% volume equalling 100 μL of the total volume was used for DNA extraction.

### 2.3 Methylation analysis

*FAM19A4/miR124‐2* methylation analysis including sample DNA extraction was performed as previously described.^24^ For bisulfite‐conversion, the EZ DNA Methylation Kit was used according to the manufacturer's specifications (Zymo Research, Irvine, California).^32^ Bisulfite‐converted DNA was subsequently used as input for quantitative PCR analysis of the *FAM19A4 and miR124‐2* promoter methylation levels using the QIAsure Methylation Test (QIAGEN). For all centers, a sample input of 2.5 μL bisulfite‐converted DNA was used for PCR on the Rotor‐Gene Q MDx 5plex HRM instrument (QIAGEN) equipped with the AssayManager software (QIAGEN). This software runs the assay followed by automatic quality assurance and data analysis. The reported results were hypermethylation‐positive, negative or invalid. Additional quality assurance was employed using the housekeeping gene β‐actin (ACTB) as a reference for successful bisulfite‐conversion, sample quality and signal normalization. Methylation testing was performed by local technicians blinded for the clinical data.

### 2.4 Data and statistical analysis

Data were analyzed per parent institute as well as combined for a pooled analysis. To assess between‐country heterogeneity we compared the prevalence, obtained clinical sensitivity and specificity between centers using chi‐square test or Fisher's exact test (where expected cell counts were below 5). In case the overall test showed a significant difference between the four centers, post hoc tests for pairwise comparison of countries were performed using a Bonferroni correction. The 95% (exact) CI's were determined for the proportions of methylation‐positive samples. Bayesian analysis was used to estimate the posterior mean and 95% credible interval of absolute risk of disease for methylation/cytology strata. The model assumes a binomial distribution for the number of patients with disease in each of the subgroups and independent noninformative uniform priors for the absolute risks. Bayesian analysis was performed in R version 3.5.3. All other analyses were performed in Stata version 14. A two‐sided significance level of 5% was used for the heterogeneity analyses.

## 3 RESULTS

The cohorts evaluated for *FAM19A4/miR124‐2* methylation comprised a total of 2656 hrHPV positive women (Table 1). Of these, 272 (10%) had invalid methylation analysis and were excluded, resulting in 2384 unique, valid samples. The mean age of women with included samples were 40.7 years, range 29 to 76 years, with the youngest mean (38 years) in Slovenia and the oldest in Denmark (52 years). A total of concurrent 2152 cytology evaluations were retrieved; 1539 normal cytology (Negative for Intraepithelial Lesions or Malignancy; NILM), 216 with Atypical Squamous Cells of Undetermined Significance (ASCUS), 166 with Low grade Squamous Intraepithelial Lesions (LSIL), and 231 with High grade Squamous Intraepithelial Lesions (HSIL) (Table 1). All centers combined, a total of 899 histologies were retrieved. Of these 527 showed no disease or CIN1 (≤CIN1), 124 (5.2%) showed CIN2, 228 (9.6%) showed CIN3 and 20 (0.8%) had cervical cancers (CC, Center‐specific frequencies in Table 1). In total, 2012 (84.4%) out of 2384 women were classified as ≤ CIN1 (consisting of women with no histology, normal histology or CIN1 histology). Centers showed heterogeneity in terms of the distribution of the women over the four categories (≤ CIN1, CIN2, CIN3, CC, Fisher's exact test *P* < .001). Post hoc pairwise comparisons showed distribution for Slovenia to differ significantly from that for Scotland, Denmark and the Netherlands (Bonferroni corrected *P*‐values for Fisher's exact test <.001, <.001 and .001, respectively). No significant pairwise differences were found between Scotland, Denmark and the Netherlands.

The combined clinical sensitivity of *FAM19A4/miR124‐2* methylation for cervical cancer was 95% (n = 20; 95%CI: 71‐99) varying between 88% and 100% (Table 2). For CIN3, the overall sensitivity was 77% (n = 228; 95%CI: 71‐82). The CIN3 sensitivity was uniform between centers varying from 75.0% (Scotland) to 78.2% (the Netherlands). For CIN2, sensitivity varied between the four centers from 33.3% (Scotland) to 61.1% (Denmark). Overall specificity of *FAM19A4/miR124‐2* methylation test among hrHPV positive women was 78.3% (n = 2012); 95%CI: 76‐80) varying from 71.1% (Scotland) to 80.3% (the Netherlands). Overall, specificity and sensitivities for CC, CIN3 and CIN2 were not found to differ between the centers (chi‐square, *P* = .10, *P* = .28, *P* = .98 and *P* = 1.0, respectively).

**TABLE 2:** Sensitivity, specificity, PPV and NPV of methylation testing; Overall and by participating center

	Specificity	Sensitivity^a^	Sensitivity^b^	Sensitivity^b^	PPV			NPV		
	(≤CIN1)	CIN2	CIN3	CC	≥CIN2	≥CIN3	CC	≥CIN2	≥CIN3	CC
All centers combined	1575/2012	58/124	176/228	19/20	253/690	195/690	19/690	1575/1694	1641/1694	1693/1694
	78.3	46.8	77.2	95.0	36.7%	28.3%	2.8%	93.0%	96.9%	99.9%
	(76.4, 80.0)	(38.1, 56.6)	(71.3, 82.2)	(70.7, 99.3)	(33.2, 40.3)	(25.0, 31.7)	(1.8, 4.3)	(91.7, 94.1)	(95.9, 97.6)	(99.6, 100.0)
Scotland	86/121	5/15	15/20	5/5	25/60	20/60	5/60	86/101	96/101	101/101
	71.1	33.3	75.0	100	41.7%	33.3%	8.3%	85.1%	95.0%	100.0%
	(62.3, 78.5)	(14.1, 60.3)	(51.5, 89.4)	(47.8, 100)***	(29.9, 54.4)	(22.6, 46.1)	(3.5, 18.5)	(76.7, 90.9)	(88.6, 97.9)	(96.4, 100.0)
Denmark	227/349	11/18	37/49	7/8	55/127	44/127	7/127	277/297	284/297	296/297
	79.4	61.1	75.5	87.5	43.3%	34.6%	5.5%	93.3%	95.6%	99.7%
	(74.8, 83.3)	(37.2, 80.7)	(61.5, 85.6)	(42.7, 98.5)	(35.0, 52.0)	(26.9, 43.3)	(2.7, 11.1)	(89.8, 96.6)	(92.6, 97.4)	(97.7, 99.9)
Slovenia	637/826	22/42	45/58	2/2	69/258	47/258	2/258	637/670	657/670	670/670
	77.1	52.4	77.6	100	26.7%	18.2%	0.8%	95.1%	98.1%	100.0%
	(74.1, 79.9)	(37.3, 67.0)	(65.0, 86.6)	(15.8, 100)***	(21.7, 32.5)	(14.0, 23.4)	(0.2, 3.0)	(93.2, 96.5)	(96.7, 98.9)	(99.4, 100.0)
The Netherlands	575/716	20/49	79/101	5/5	104/245	84/245	5/245	575/626	604/626	626/626
	80.3	40.8	78.2	100	42.4%	34.3%	2.0%	91.9%	96.5%	100.0%
	(77.2, 83.0)	(28.0, 58.1)	(69.1, 85.2)	(48.8, 100)***	(36.4, 48.7)	(28.6, 40.4)	(0.9, 4.8)	(89.4, 93.8)	(94.7, 97.7)	(99.4, 100.0)

*Note*: 95% confidence intervals based on logit transformation except *** one‐sided 97.5% exact confidence intervals.

a Calculated as % women in group with no histology, normal histology or CIN1 histology that were methylation‐negative.

b Calculated as % women in histology group (CIN2, CIN3 or CC) that were methylation‐positive.

The negative predictive value of hrHPV positive, methylation‐negative outcomes were 99.9% for cervical cancer (N = 1694; 95%CI: 99.6‐100, 96.9% for ≥CIN3 (95%CI: 96‐98), and 93.0% for ≥CIN2 (95%CI: 92‐94). The overall positive predictive value (PPV) of hrHPV positive, methylation‐positive outcomes for ≥CIN3 was 28.3% (N = 690, 95%CI: 25‐32) and 36.7% for ≥CIN2 (95%CI: 33‐40). When methylation was stratified by HSIL cytology, the sensitivity for CIN3 was 85.2% (95%CI: 78%‐90%) and specificity was 46% (95%CI: 29%‐63%; Table 3). For ASCUS and LSIL, sensitivities for CIN3 were lower at 75.0% (95%CI: 57%‐87%) and 68.2% (95%CI: 46%‐84%), respectively. Specificities were 70.1% (95%CI: 62%‐77%) and 85.2% (95%CI: 78%‐91%), respectively. For NILM, CIN3 sensitivity was 27.8% (95%CI: 12%‐53%) with a specificity of 79.0% (95%CI: 77%‐81%). A complete reporting by participating center can be found in Supporting Information Table 1.

**TABLE 3:** Specificity and sensitivity of methylation testing stratified by cytology grade for all centers combined

Cytology grade (N, %, range)	Specificity	Sensitivity	Sensitivity	Sensitivity
	(≤ CIN1)	CIN2	CIN3	CC
NILM cytology	1183/1497	6/22	5/18	2/2
	79.0	27.3	27.8	100
	(76.9, 81.0)	(12.5, 49.5)	(11.7, 52.7)	(15.8, 100)*
ASCUS	108/154	12/25	24/32	5/5
	70.1	48.0	75.0	100
	(62.4, 76.9)	(29.3, 67.3)	(57.1, 87.1)	(47.8, 100)*
LSIL	99/115	12/28	15/22	1/1
	85.2	42.9	68.2	100
	(77.5, 90.6)	(25.9, 61.7)	(46.1, 84.3)	(2.5, 100)*
HSIL	15/33	26/45	121/142	10/11
	45.5	57.8	85.2	90.9
	(29.4, 62.6)	(42.9, 71.3)	(78.3, 90.2)	(53.6, 98.9)

*Note*: 95% confidence intervals based on logit transformation except *one‐sided 97.5% exact confidence intervals.

**FIGURE 1:**
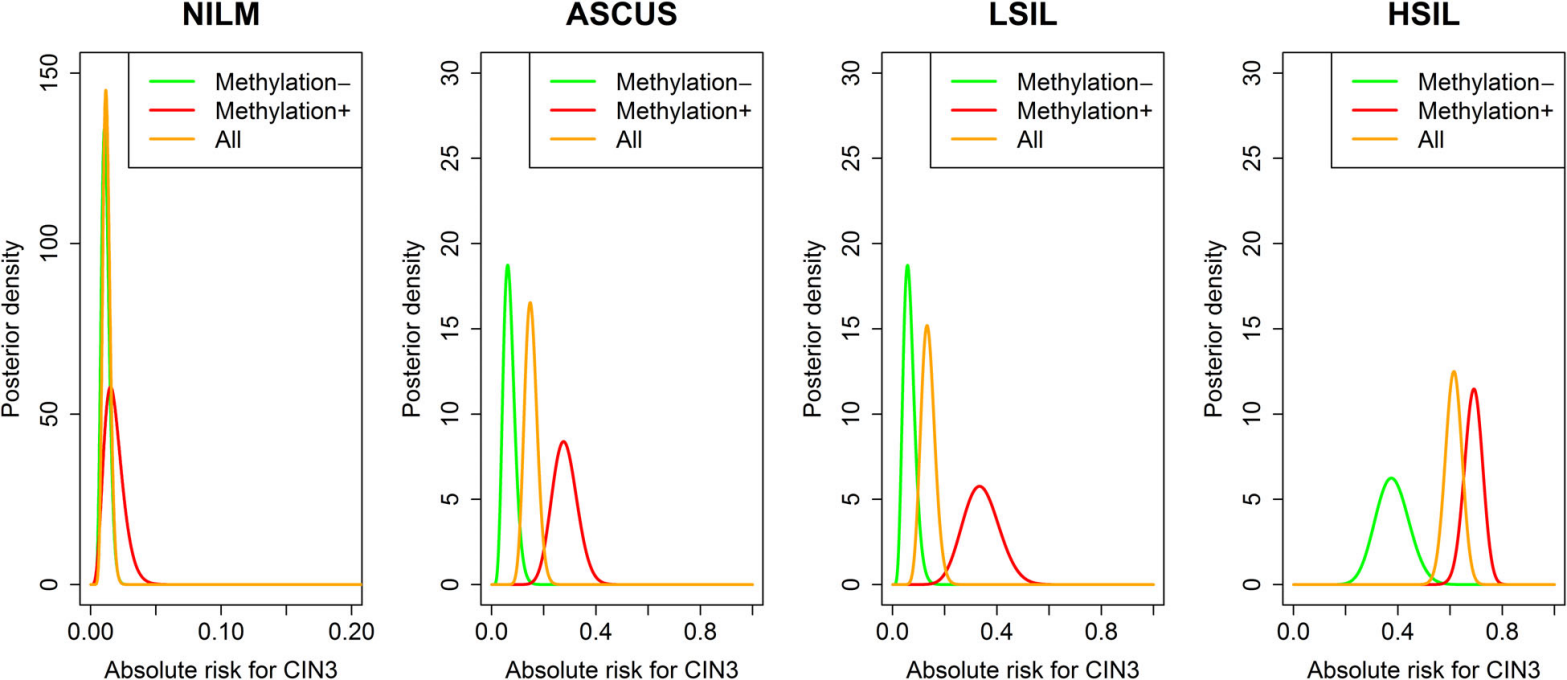
Absolute risk plots for CIN3 by hrHPV positive and FAM194A/miR124‐2 methylation‐positive cervical screening samples stratified by concurrent cytology

We employed a Bayesian analysis to determine the risk of CIN3 for hrHPV and methylation‐positive samples with concurrent NILM to be 1.5% (95%CI: 0.5%‐3.5%, Table 4), 27.6% for ASCUS (95%CI: 19%‐38%), 33.3% for LSIL (95%CI: 20%‐49%), and 69.1% for HSIL (95%CI: 62%‐76%). Absolute risk plots for CIN3 by hrHPV positive and *FAM194A/miR124‐2* methylation‐positive cervical screening samples stratified by concurrent cytology shows the largest discriminating power of *FAM194A/miR124‐2* methylation to be in the LSIL and ASCUS classes displaying highly comparable distributions (Figure 1).

**TABLE 4:** Risk of CIN3 by cytology and methylation

Cytology (N, %, range)	Methylation−	Methylation+	All
NILM	13/1212	5/327	18/1539
	1.0	1.5	1.2
	(0.6, 1.8)	(0.5, 3.5)	(0.7, 1.8)
ASCUS	8/129	24/87	32/216
	6.2	27.6	14.8
	(2.7, 11.9)	(18.5, 38.2)	(10.4, 20.3)
LSIL	7/121	15/45	22/166
	5.8	33.3	13.3
	(2.8, 11.7)	(20.0, 49.0)	(8.9, 19.3)
HSIL	21/56	121/175	142/231
	37.5	69.1	61.5
	(24.9, 51.5)	(61.7, 75.9)	(54.9, 67.8)

*Note*: 95% credible interval based on binomial likelihood and uniform prior.

## 4 DISCUSSION

Effective triage of hrHPV positive screening samples constitutes one of the currently most crucial scientific issues to solve for primary HPV based screening to truly modernize cervical cancer prevention. As long as cytology remains the triage of choice, only countries with high quality cytology can operate a relatively well‐balanced HPV screening in terms of screening detection vs overreferral. But the high degree of reliance upon the subjective skills of cytologists remains and retention and recruitment of this skilled workforce is increasingly challenged.

This large multicenter retrospective clinical performance study shows that triage of HPV‐positive women with the *FAM19A4/miR124‐2* methylation test yields objective, reproducible results in terms of ≥CIN3 detection across four European countries using different cervical sample collection medias, DNA extraction methods and HPV screening tests. Our data show that the test has a high sensitivity for cervical cancer and CIN3 (ie, 95% and 77.2%, respectively) with good specificity (ie, 78.3%) among HPV‐positive women. Our study represents the largest clinical evaluation of a defined methylation biomarker panel to date. The results confirm and expand earlier studies with these biomarkers in HPV‐positive women, as well as the earlier described high assay reproducibility.^19^, ^23^, ^24^, ^25^

A key observation from our study is that 19 of 20 screen‐detected cervical cancers were found methylation‐positive (Table 2). The one negative carcinoma (stage 1A, HPV31 and HPV52 positive) in our study had methylation levels close to, yet below the defined assay cut off. Retesting showed methylation assay outcomes either above or below the threshold (data not shown). The current data therefore supports our previous findings from a large retrospective study across five continents reporting that the *FAM19A4/miR124‐2* methylation assay detected >98% of cervical cancers, independent of histology type, geographical area, sample type and HPV genotype.^25^ Together, this leads us to propose that methylation of *FAM19A4/miR124‐2* appears to be a rather universal event in cervical carcinogenesis and that women testing methylation‐negative are unlikely to have a prevalent cancer. In addition, post hoc analyses of a large screening trial by De Strooper et al^19^ and Dick et al^20^ have shown that the long‐term risk of hrHPV positive, *FAM19A4/miR124‐2* methylation‐negative women is similar or better for cervical cancer and ≥CIN3 endpoints compared to a negative cytology test. This implies that 3‐year to 5‐year screening intervals can be maintained with safety for HPV based screening with a negative *FAM19A4/miR124‐2* methylation triage test.

A remarkable finding was that despite the variation in cervical sample media, primary HPV assay, and DNA extraction method between the participating centers, the sensitivity for CIN3 was very stable at 77% with only small variability between centers (range, 75%‐78%; Table 2). In the more heterogeneous CIN2 group, the clinical sensitivity showed more variation between centers (range, 33%‐61%, Table 2). Such a variation in clinical sensitivity for CIN3 and CIN2 was reported previously on a panel of 12 methylation markers showing a cancer‐like methylation patterns in 72% of CIN3 and 55% of CIN2,^33^ confirming the robustness of methylation testing in detecting advanced CIN, but also attesting to the well‐known fact that the diagnosis of CIN2 based upon hematoxylin and eosin (H&E)‐stained cervical biopsies is subject to substantial interobserver variability.^34^ An important feature of operationalization is the robustness of an analysis, and here we observed variations in the methylation assay invalid rates between centers. However, since all included samples were retrieved from biobanks, the age and storage of samples could play a role in the variable invalid rates.

For a period, cytology will remain the triage method of choice in primary screening. However, HPV‐positive women with ASCUS and LSIL generate large numbers of repeat tests and/or unnecessary colposcopy referrals while detecting relatively few ≥CIN3. In this context, direct triage using *FAM19A4/miR124‐2* methylation could substitute cytology or be used in combination with cytology and thereby lead to a 66% reduction in colposcopy referrals in these women without loss of clinical sensitivity. Figure 1 shows that the highest distinguishing value of the *FAM19A4/miR124‐2* methylation assay is in hrHPV positive women with concurrent ASCUS and LSIL. On the other hand, the diagnosis of HSIL carries such high risk of CIN3 that these women should be sent to colposcopy regardless of methylation status (Figure 1), even though the risk of CIN3 was markedly lower for HSIL, methylation‐negative cases compared to the HSIL, methylation positive cases.

A limitation of our large‐scale multicenter study is a verification bias for cytology because the majority of included samples were referred after abnormal cytology making a head‐to‐head comparison between cytology and methylation impossible. With only 18 ≥CIN3 cases after NILM cytology, the relative sensitivity of the *FAM19A4/miR124‐2* methylation test would be underestimated. Another limitation of our study relates to the invalid rates observed. With invalid rates of 7.5% (Scotland and Slovenia), 3% (the Netherlands) and 24% (Denmark), invalid methylation samples could at first glance seen to be an issue for routine implementation. A driving factor for invalid test results were low‐cellularity/DNA, yet samples could in general not be repeated routinely with more sample input for example, 4× to 10× more as this was not available given the retrospective nature of our study using biobanked and residual material from completed routine screening. Looking into methylation testing reproducibility, we have previously shown a high degree of this as presented in Floore et al.^24^ Of a more technical nature, the bisulfate conversion step required local optimization within the confines of the manufacture's specifications to obtain the best assay performance rate. After undergoing optimization, invalid rate in the final testing rounds of the Danish samples reached 6%. Finally, newer bisulfate conversion kits have been marketed since this work, and it remain to be investigated whether this new generation conversion chemistry will lead to significant improvements in resulting test quality. In conclusion, it seems that because of the study design the number of invalids is overestimated compared to routine diagnostic settings.

Statistical comparisons revealed a significant lower PPV for ≥CIN3 in Slovenia compared to the Netherlands (Bonferroni corrected *P* < .001, data not shown), and a trend toward a difference between Denmark and Slovenia (Bonferroni corrected *P* = .051, data not shown). Yet, the PPV depends on the prevalence of CIN lesions, which was found to be lower in Slovenia compared to the other countries. Since detection of CIN lesions is based on the diagnosis of abnormal cytology which—especially for low grade cytology—shows limited reproducibility, these differences in cytology scoring may account for some of the differences observed between centers. Other factors affecting the PPV in our study are screening history. Here, Slovenia screens most often at 3 years, Denmark and Scotland in between at 3‐year and 5‐year intervals, with the Netherlands having 5‐year intervals. Finally, as PPV is also strongly associated to age, the mean age difference between the cohorts could also have influenced the PPV.

The implications of molecular biomarkers for triage in cervical cancer screening practice are considerable. Today, all HPV based screening programs rely on (repeat) cytology as triage test for colposcopy referral, with or without HPV16/18 genotyping. Yet, cytology is largely subjective in its nature and leads to overreferral especially for low‐grade cellular abnormalities and therefore remains a challenge from the clinical perspective. Substituting cytology in triage of hrHPV positive screening samples with an objective molecular biomarker test with a high PPV for ≥CIN2 or ≥CIN3 such as *FAM19A4/miR124‐2* methylation, colposcopy referrals can be markedly reduced while maintaining good sensitivity for cervical cancer and advanced CIN. The reassurance that cancer is most likely not present after a negative methylation test result supports surveillance rather than referral, to the benefit of all women participating in^3^screening.

## 5 CONCLUSION

The *FAM19A4/miR124‐2* methylation test shows a high sensitivity and PPV for ≥CIN3 as a colposcopy triage test of HPV‐positive women exceeding the PPV thresholds for colposcopy referral in the US (cut off for referral 10%) and many other western countries (10%‐20%). A further advantage is the objective machine read results based upon a clinical cut off which will not be influenced that is, by HPV vaccination status. Since the long‐term safety of the *FAM19A4/miR124‐2* methylation test is equal for ≥CIN3^20^ and in this series was even higher for cervical cancer compared to cytology, the use of this methylation assay as a triage marker shows promise. Translated into clinical practice this would potentially lead to lower colposcopy referrals and less retests elicited by hrHPV positive screening samples. Finally, the retest period could safely be extended to 3 years as was recently proposed for hrHPV positive women with low grade cytology triage outcomes.^35^ In perspective, these conclusions support practical pilot implementation of the *FAM19A4/miR124‐2* methylation test into cervical screening program's to provide further data and experiences to inform on how a fully molecular cervical screening program can be designed to the benefit of women and health care services both.

## CONFLICT OF INTERESTS

The project was funded by the SME Instrument of the European Commission in the HORIZON2020 (Valid‐screen contract ID: 666800).

Jesper Bonde's institution has received research funding or consumables at reduced price or for free to support research from BD Diagnostics, Agena Bioscience, Genomica SAU, LifeRiver Biotech and QIAGEN. He has received honoraria for lectures from BD Diagnostics and Hologic Ltd. Jesper Bonde is an appointed member of the National Danish Cervical Screening Committee by the Danish Health Authority, and a member of the cervical screening steering committee of the Capital Region of Denmark.

Kate Cuschieri institution has received research funding or gratis consumables to support research from the following commercial entities in the last 3 years: Cepheid, Genomica, LifeRiver, Euroimmun, GeneFirst, SelfScreen, Qiagen, Hiantis and Hologic.

Grazyna Stanczuk received diagnostic tests from Roche and travel sponsorship and speaker's fee from Roche and Abbott. DAMH has been on the speaker's bureau of QIAGEN, serves occasionally on the scientific advisory board of Pfizer and Bristol‐Meyers Squibb, and has minority stake in Self‐screen B.V., a spin‐off company of VU University Medical Center (currently known as Amsterdam UMC, Vrije Universiteit Amsterdam). Self‐screen B.V. develops, manufactures and licenses the high‐risk HPV assay and methylation marker assays for cervical cancer screening and holds patents on these tests.

Chris J. L. M. Meijer is minority shareholder and part‐time CEO of Self‐screen B.V., a spin‐off company of VUmc, which develops, manufactures and licenses the high‐risk HPV assay and methylation marker assays for cervical cancer screening and holds patents on these tests. CJLMM has a very small number of shares of QIAGEN and MDXHealth, has received speakers' fees from GSK, QIAGEN, and SPMSD/Merck, and served occasionally on the scientific advisory boards (expert meeting) of these companies.

Anja Oštrbenk Valenčak has received reimbursement of travel expenses for attending conferences and honoraria for speaking from Abbott Molecular, Qiagen and Seegene.

Arno Floore, Saskia Doorn and Albertus Hesselink are employed by Self‐screen B.V.

All other authors report no conflict of interest.

## ETHICS STATEMENT

The work in our study with human derived material was conducted under national and international rules and legislation, as well as European standards of research ethics, as it is expressed in the applicable legislation/regulations (The Declaration of Helsinki; informed consent for participation of human subjects in medical and scientific research) and guidelines for Good Clinical Practice. The study was approved by the local ethics committees where applicable. The Danish data contribution: Danish Data Protection Agency AHH 2015‐085, I‐suite: 04150 and AHH‐2017‐024, I‐Suite: 05356.

## Supporting information


**Data S1** Supplementary Information.

## Data Availability

Data in anonymized form can be made available upon reasonable request to the senior author, and following the Danish, Slovenian, Scottish and Dutch Data Protection Regulation.
