# Supplementary material for: Methylation markers FAM19A4 and miR124‐2 as triage strategy for primary human papillomavirus screen positive women: A large European multicenter study
Source: Int J Cancer. 2020 Oct 21;148(2):396–405. doi: 10.1002/ijc.33320 (PMC7756277; doi:10.1002/ijc.33320)
Supplement: Supplementary file 1 — Data S1 Supplementary Information. [file IJC-148-396-s001.pdf]

Supplementary material
Methylation markers *FAM19A4* and *miR124-2* as triage strategy for primary HPV screen positive women; A large European multi-center study

Jesper Bonde[1§], Arno Floore[2], Ditte Ejegod[1], Frederique J. Vink[8], Albertus Hesselink[2], Peter M. van de Ven[10], Anja Oštrbenk Valenčak[5], Helle Pedersen[1], Saskia Doorn[2], Wim G.Quint[7], K. Ulrich Petry[4*], Mario Poljak[5], Grazyna Stanczuk[9], Kate Cuschieri[3], Silvia de Sanjosé[6], Maaike Bleeker[8], Johannes Berkhof[10], Chris J.L.M. Meijer[2,8], Daniëlle A.M. Heideman[8]

Table of contents

Supplementary Table 1: Specificity and sensitivity of methylation testing per cytology grade stratified by center

Supplementary Table 1 Specificity and sensitivity of methylation testing per cytology grade stratified by center

normal cytology	Specificity (≤ CIN1)	Sensitivity CIN2	Sensitivity CIN3	Sensitivity CC
**All centers combined (N, %, Range)**	1192/1497<br>79.1<br>(77.7, 81.1)	6/22<br>27.3<br>(12.5, 49.5)	5/18<br>27.8<br>(11.7, 52.7)	2/2<br>100<br>(15.8, 100)*
**Scotland**	71/99<br>71.7<br>(62.0, 79.7)	1/5<br>20.0<br>(2.1, 74.4)	1/4<br>25.0<br>(2.4, 82.0)	N/A
**Denmark**	40/45<br>88.9<br>(75.7, 95.3)	N/A	N/A	N/A
**Slovenia**	544/708<br>76.8<br>(73.6, 79.8)	1/4<br>25.0<br>(2.4, 82.0)	1/4<br>25.0<br>(2.4, 82.0)	N/A
**The Netherlands**	528/645<br>81.9<br>(78.7, 84.7)	4/13<br>30.7<br>(11.5, 60.2)	3/10<br>30.0<br>(9.3, 64.1)	2/2<br>100<br>(15.8, 100)*
**ASCUS cytology**				
**All centers combined (N, %, Range)**	108/154<br>70.1<br>(62.4, 76.9)	12/25<br>48.0<br>(29.3, 67.3)	24/32<br>75.0<br>(57.1, 87.1)	5/5<br>100<br>(47.8, 100)*
**Scotland**	9/14<br>64.3<br>(36.7, 84.8)	0/1<br>0<br>(0, 97.5)[3]	1/1<br>100<br>(2.5, 100)[3]	4/4<br>100<br>(39.8, 100)*
**Denmark**	20/47<br>63.8<br>(49.2, 76.3)	3/4<br>75.0<br>(18.0, 97.6)	5/8<br>55.6<br>(23.6, 83.5)	N/A

**Slovenia**	38/51<br>74.5<br>(60.7, 84.7)	3/8<br>37.5<br>(11.5, 73.5)	7/9<br>77.8<br>(39.8, 94.9)	1/1<br>100<br>(2.5, 100)*
**The Netherlands**	31/42<br>73.8<br>(58.4, 85.0)	6/12<br>50.0<br>(23.4, 76.5)	11/14<br>78.6<br>(49.3, 93.2)	N/A
**LSIL cytology**				
**All centers combined (N, %, Range)**	98/115<br>85.2<br>(77.5, 90.6)	12/28<br>42.9<br>(25.9, 61.7)	15/22<br>68.2<br>(46.1, 84.3)	1/1<br>100<br>(2.5, 100)*
**Scotland**	4/5<br>80.0<br>(25.6, 97.9)	1/4<br>25.0<br>(2.4, 82.0)	2/2<br>100<br>(15.8, 100)*	1/1<br>100<br>(2.5, 100)*
**Denmark**	36/39<br>92.3<br>(78.4, 97.5)	1/1<br>100<br>(2.5, 100)*	1/1<br>100<br>(2.5, 100)*	N/A
**Slovenia**	48/54<br>88.9<br>(77.2, 95.0)	7/10<br>70.0<br>(36.0, 90.7)	4/7<br>57.1<br>(20.9, 87.0)	N/A
**The Netherlands**	10/17<br>58.8<br>(34.5, 79.5)	3/13<br>23.1<br>(7.3, 53.5)	8/12<br>66.7<br>(36.3, 87.5)	N/A
**HSIL cytology**				
**All centers combined (N, %, Range)**	15/33<br>45.5<br>(29.4, 62.6)	26/45<br>57.8<br>(42.9, 71.3)	121/142<br>85.2<br>(78.3, 90.2)	10/11<br>90.9<br>(53.6, 98.9)
**Scotland**	1/2<br>50.0<br>(1.9, 98.1)	3/5<br>60.0<br>(16.9, 91.8)	11/13<br>84.6<br>(53.4, 96.4)	N/A

**Denmark**	3/12<br>25.0<br>(7.8, 56.6)	5/9<br>55.6<br>(23.6, 83.5)	21/27<br>77.8<br>(58.1, 89.8)	6/7<br>85.7<br>(37.8, 98.3)
**Slovenia**	7/13<br>53.8<br>(27.3, 78.4)	11/20<br>55.0<br>(33.1, 75.1)	33/38<br>86.8<br>(71.8, 94.5)	1/1<br>100<br>(2.5, 100)*
**The Netherlands**	2/6<br>66.7<br>(23.7, 92.8)	7/11<br>63.6<br>(32.5, 86.4)	56/64<br>87.5<br>(76.8, 93.7)	3/3<br>100<br>(29.2, 100)*

95% confidence intervals based on logit transformation except *one-sided 97.5% exact confidence intervals.
